# A solid state fungal fermentation-based strategy for the hydrolysis of wheat straw^☆^

**DOI:** 10.1016/j.biortech.2013.09.061

**Published:** 2013-12

**Authors:** Nattha Pensupa, Meng Jin, Matt Kokolski, David B. Archer, Chenyu Du

**Affiliations:** a School of Biosciences, Sutton Bonington Campus, University of Nottingham, Loughborough, Leicestershire LE12 5RD, United Kingdom; b School of Life Sciences, University Park, University of Nottingham, Nottingham NG7 2RD, United Kingdom

**Keywords:** Solid state fermentation (SSF), Wheat straw, *Aspergillus niger*, Cellulase, Hydrolysis

## Abstract

This paper reports a solid-state fungal fermentation-based pre-treatment strategy to convert wheat straw into a fermentable hydrolysate. *Aspergillus niger* was firstly cultured on wheat straw for production of cellulolytic enzymes and then the wheat straw was hydrolyzed by the enzyme solution into a fermentable hydrolysate. The optimum moisture content and three wheat straw modification methods were explored to improve cellulase production. At a moisture content of 89.5%, 10.2 ± 0.13 U/g cellulase activity was obtained using dilute acid modified wheat straw. The addition of yeast extract (0.5% w/v) and minerals significantly improved the cellulase production, to 24.0 ± 1.76 U/g. The hydrolysis of the fermented wheat straw using the fungal culture filtrate or commercial cellulase Ctec2 was performed, resulting in 4.34 and 3.13 g/L glucose respectively. It indicated that the fungal filtrate harvested from the fungal fermentation of wheat straw contained a more suitable enzyme mixture than the commercial cellulase.

## Highlights

• A solid state fungal fermentation strategy converting wheat straw to hydrolysate.
• A biological pre-treatment of wheat straw by culturing *A. niger* on wheat straw.
• 24.0 ± 1.76 U/g cellulase was produced using wheat straw as the main substrate.
• The fungal extract was used to hydrolyze the fermented wheat straw.
• 19% higher hydrolysis efficiency using freshly-prepared fungal extract than Ctec2.

## 1 Introduction

There is an increasing interest in the production of biofuels as an alternative solution to the energy shortage and greenhouse gas emission. In 2011, around 85 billion litres of bioethanol were produced worldwide. Currently, bioethanol is predominately synthesised via the 1st generation production process, where food-based crops such as corn, sugar cane and wheat are used as the starting material. However, converting food materials to biofuel triggered the concern of global food security and that significantly affected the public acceptance of biofuel. Therefore, research into biofuels has been focusing on the development of advanced generations of biofuels. Within these non-food biofuel processes, production of bioethanol from lignocellulosic raw materials, e.g. wheat straw, corn stover and bagasse (Singh and Bishnoi, 2012; Avci et al., 2013) are promising options.

The lignocellulosic bioethanol process is still yet to be commercialized at an industrial scale. One of the key challenges is the generation of fermentable sugars from lignocellulosic raw materials. Generally, a pre-treatment process is required, followed by an enzymatic hydrolysis process catalyzed by cellulases and other glycosyl hydrolases (GHs). Various pre-treatment methods, e.g. dilute acid pre-treatment, alkali pre-treatment, steam explosion, supercritical CO_2_ explosion were developed and have been applied on a broad range of biomass raw materials. Although these pre-treatment methods reduced biomass recalcitrance and increased surface area (Ibbett et al., 2011), they are energy intensive and release compounds that may inhibit subsequent fermentation. Therefore, an efficient pre-treatment process is still yet to be developed.

Wheat (*Triticum aestivum* L.) is the world’s most widely grown crop, cultivated in over 115 nations. In Europe, wheat is the dominant cereal crop. The utilisation of wheat grains for the production of bioethanol and biochemicals has been well studied. One promising process in wheat biorefining strategies is to grow fungi directly on the wheat and wheat milling by-products for the production of hydrolytic enzymes (Dorado et al., 2009; Du et al., 2008). These enzymes then hydrolyze the starch and fungal biomass itself to generate a glucose-rich stream and a nitrogen-rich stream. Combining these two streams formed a nutrient complete medium which was then fermented to bioethanol (Koutinas et al., 2007a), succinic acid (Lin et al., 2011; 2012) and PHB (Koutinas et al., 2007b). However, this approach has focussed mainly on the starch components from the wheat grains. There is no report available on the biorefinery development using the wheat straw. On the other hand, wheat straw is the most abundant lignocellulosic raw material in Europe and the second in the world after rice straw (Kim and Dale, 2004). It is estimated that around 350 million tons of wheat straw are available annually worldwide for the production of bioethanol (Sarkar et al., 2012), which could generate around 100 billion litres of bioethanol. This indicates the great potential of wheat straw as a promising renewable biomass. The composition of wheat straw changes according to wheat variety, season, soil condition and harvest time. Generally, dry wheat straw is composed of cellulose 35–45%, hemicellulose 20–30%, lignin 8–15%, protein 3.1% and ash 10.1% (Sarkar et al., 2012). Wheat straw has been widely used as a model lignocellulosic raw material for the exploration of an efficient pre-treatment method, including dilute acid pre-treatment, ionic liquid, steam explosion, hydrothermal and biological pre-treatment. These studies have been recently reviewed by Talebnia et al. (2010) and Sarkar et al. (2012).

**Fig. 1:**
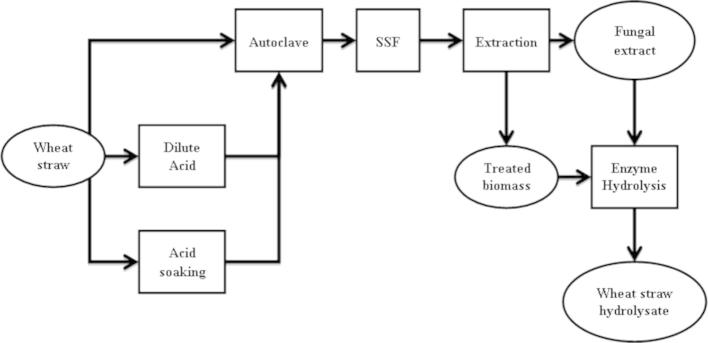
The schematic diagram of wheat straw-based biorefining processes.

In this paper, we report a wheat straw-based biorefining strategy. In the first stage, wheat straw was exposed to *A. niger* in solid state fungal fermentation for the production of cellulosic enzymes. In the second stage, the enzyme mixture was extracted and then used to hydrolyze the fermented wheat straw. A schematic diagram of the process is shown in Fig. 1.

## 2 Methods

### 2.1 Wheat straw

Cordiale wheat straw (*Triticum aestivum* L.) was obtained from the University Farm (University of Nottingham, Sutton Bonington, UK). The air-dried wheat straw was cutting-milled and passed through a 2-mm screen sieve (Fritsch, Idar-Oberstein, Germany). The wheat straw was collected and stored at cold room until use.

### 2.2 Microorganisms

*Aspergillus niger* N402 was used in solid state fungal fermentation. Procedures for storing, and cultivating *A. niger* were described by Delmas et al. (2012).

### 2.3 Modification of wheat straw

Both crude wheat straw and modified wheat straw were used in this study. The following methods were used to modify the wheat straw.

*Autoclave modification:* after adjusting the moisture content to the designed water to wheat straw ratio, the wet wheat straw was autoclaved at 121 °C for 15 min.

*Dilute acid modification:* Wheat straw was mixed with 1%, 2%, 3% (v/v) H_2_SO_4_ or HCl solution, respectively, at a solid to liquid ratio of 1:10 (w/v), autoclaved at 121 °C for 30 min. The autoclaved wheat straw was adjusted to pH 7 by 1 M NaOH solution and then rinsed with distilled water to remove acid residues. Collected biomass was dried in an oven at 40 °C until the weight was constant.

*Acid soaking modification:* wheat straw was mixed with 1% H_2_SO_4_ solution at a solid to liquid ratio of 1:10 (w/v), incubated in a water bath at 50 °C for 30 min. The treated wheat straw was adjusted to pH 7 by 1 M NaOH solution and then rinsed off with distilled water to remove acid residue. Collected biomass was dried in an oven at 40 °C until the weight was constant.

### 2.4 Solid state fermentation (SSF), media and conditions

Each 16 g (dry weight) of crude wheat straw or the modified wheat straw, as described above, was weighed and put into 250 ml Duran bottles. The impact of the moisture content on cellulase production in the SSF was examined. Distilled water was added to the wheat straw to adjust the liquid to solid ratio to 5:1, 6:1, 7:1, 7.5:1, 8:1 and 9:1 (w/w), equating to moisture contents 85.1%, 87.2%, 88.8%, 89.5%, 90.1% and 91.1%, respectively. Starch powder (2 g/L) was added to improve the fungal growth. The Duran bottles were sterilized at 121 °C for 15 min. SSF experiments were commenced by adding spore suspension (1 × 10^6^ spore/g of dry wheat straw) into each Duran bottle. A sterilized spatula was used to mix the mash in the Duran bottle to enable even distribution of the inoculum. Around 2 g of inoculated wheat straw mash were distributed to each Petri dish and then incubated in a static incubator at 28 °C for up to 7 days.

In the experiments of investigating the impact of additional nutrients on the cellulolytic enzyme production, 0.5% or 5% (w/v) yeast extract was added to the wheat straw substrate before autoclaving. In the experiments of adding a mineral solution, the following mineral solution was used to replace distilled water (L^−1^): (NH_4_)_2_SO_4_ 1 g, KH_2_PO_4_ 0.5 g, K_2_HPO_4_ 0.5 g and MgSO_4_ 0.2 g. All the SSF were carried out in triplicate at least. Data means and standard deviations were calculated using Microsoft Excel. Student’s *t*-test was performed at the level of *p*-value (<0.05) to determine the significance of the different experimental group.

### 2.5 Enzyme extraction

The fermented wheat straw mash from each Petri dish was transferred into a blender (Waring commercial blender BN974, USA). Then, 30 ml of 50 mM citric acid buffer per Petri dish was added. The mash was then blended at “high” power for 10 s. The mixture was transferred to a beaker and the contents were stirred at 4 °C, 300 rpm for 30 min. Then the mixture was centrifuged at 5000 rpm (4696*g*) for 10 min. The clear supernatant (fungal extract) was used as the crude enzyme mix. In the moisture content experiments, no blender was used. The fermented wheat straw mash was directly transferred to a beaker containing 30 ml of 50 mM citric acid buffer per Petri dish. It was then stirred by a magnetic stirrer 4 °C, 300 rpm for 30 min. After centrifugation as above, the clear supernatant was collected as the crude enzyme.

### 2.6 Enzyme assay

#### 2.6.1 Cellulase (filter paper units)

Filter paper cellulase activity was determined according to NREL Laboratory Analytical Procedure (Adney and Baker, 1996). 0.5 ml suitable diluted enzyme solution was mixed with 1 ml citric acid buffer (50 mM, pH 4.8) in a test tube containing a Whatman No. 1 filter paper strip (1.0 × 6.0 cm, around 50 mg). The reaction mixtures were incubated at 50 °C for 60 min. The filter paper activity (U/ml) was calculated using the following equation (Adney and Baker, 1996):

$$\text{Filter paper activity}\;(\text{U/ml})=\frac{0.37}{\text{Concentration of enzyme that releases}\;2.0\;\text{mg glucose}}$$

Then it was converted to U per gram of dry weight wheat straw using the following equation:

$$\text{Filter paper activity}\;(\text{U/g})=\frac{\text{Filter paper activity}\;(\text{U/ml})\times \text{total volume of the fungal extract}\;(\text{ml})}{\text{Dry weight of the wheat straw used in the SSF}\;(\text{g})}$$

#### 2.6.2 Endoglucanase

Endo-β-1,4-glucanase (carboxymethyl cellulase, CMCase) was analyzed by measuring the amount of glucose liberated from 2% (w/v) carboxymethyl cellulose solution in 0.05 M sodium citrate buffer pH 4.8, according to Ghose (1987). The assay mixture contained 0.5 ml of 2% (w/v) carboxymethyl cellulose solution and 0.5 ml of enzyme solution. This assay was carried out at 50 °C for 30 min. The reaction was stopped by adding 3 ml of DNS solution and heating the tube in a boiling water bath for 5 min. The amount of sugar released was measured by absorbance at 540 nm using a spectrophotometer.

#### 2.6.3 Exo-glucanase

Exo-1,4-β-glucanase (Avicelase) activity was measured using 0.5 ml of 1% avicel solution in 0.05 M sodium citrate buffer and 0.5 ml of enzyme solution (Ghose, 1987). This mixture was incubated at 50 °C for 30 min. The reaction was stopped by adding 3 ml of DNS solution and heating the tube in a boiling water bath for 5 min. The amount of sugar released was measured by absorbance at 540 nm using a spectrophotometer.

#### 2.6.4 β-Glucosidase

β-Glucosidase activity was measured by the method of Herr (1979). The reaction mixture contained 1 ml of 2 mM *p*-nitrophenyl-β-d-glucopyranoside (pNPG) and 0.1 ml of enzyme solution. This reaction was carried out at 50 °C for 5 min. The reaction was stopped by adding 2 ml of 1 M sodium carbonate (Na_2_CO_3_) solution. The amount of *p*-nitrophenol was determined by absorbance at 405 nm using a spectrophotometer.

#### 2.6.5 Xylanase

Xylanase activity was determined by the method of Bailey et al. (1992). 1% beech wood xylan was dissolved in 0.05 M sodium citrate buffer, at pH 4.8. The reaction mixture contained 0.5 ml of 1% beech wood xylan and 0.5 ml of enzyme solution. The reaction was carried out at 50 °C for 5 min. It was stopped by adding 3 ml of DNS solution and heating the tube in a boiling water bath for 15 min. The amount of sugar released was measured by absorbance at 540 nm using a spectrophotometer.

One international unit of enzyme activity was defined as the amount of enzyme required to release 1 μmol of product (glucose for CMCase and Avicelase, *p*-nitrophenol for β-glucosidase and xylose for xylanase) from an appropriate substrate per min under given assay conditions.

### 2.7 Hydrolysis

Three different enzyme solutions and two different substrates were used in the hydrolysis experiments. Enzyme solution 1 (Ctec2): The Ctec2 was prepared by diluting 90 μl of Novozyme Cellic® Ctec2 in 40 ml citrate buffer (50 mM, pH 4.8). The enzyme loading ratio was around 30 U/g dry biomass. Enzyme solution 2 (fungal extract): 40 ml crude fungal extract from the SSF using autoclaved wheat straw with 0.5% yeast extract and minerals (cellulase activity 24.0 U/g). The enzyme loading ratio was 30 U/g dry biomass. The fungal extract already contained the citric acid buffer. Enzyme solution 3 (1:1 Ctec2 and fungal extract) was prepared by diluting 45 μl of Novozyme Cellic® Ctec2 in 20 ml citrate buffer (50 mM, pH 4.8), then mixing with 20 ml of the same fungal extract as described above. The enzyme loading ratio was 30 U/g dry biomass. Hydrolysis substrate 1 (fermented wheat straw) contained the fermented wheat straw from the SSF using autoclaved wheat straw with 0.5% yeast extract and minerals. Hydrolysis substrate 2 (autoclaved wheat straw) contained the wheat straw autoclaved at 121 °C, 15 min.

The hydrolysis was carried out by adding 2 g (dry weight) substrate into the enzyme solution, and then the samples were shaken in a water bath at 150 rpm, 50 °C and for 3 days. Samples were collected and centrifuged at 13,000 rpm (16,249*g*) for 10 min, the sugar concentrations in the supernatant were analyzed by HPLC.

The glucose production yield was calculated by the following equation:

$$\text{Glucose production yield}\;(\text{g/g})=\frac{\text{Glucose concentration in the hydrolysate}\times \text{total volume}}{\text{Dry weight of the wheat straw added in the reaction}}$$

The glucose hydrolysis yield was calculated using the following equation:

$$\text{Glucose hydrolysis yield}\;(\text{g/g})=\frac{\text{Glucose released per g dry weight wheat straw}}{1.1\times \text{Cellulose concentration in the dry weight wheat straw}}$$

Cellulose concentration in the wheat straw was 39% (dry weight), (Ibbett et al., 2011).

### 2.8 Glucosamine assay

The glucosamine concentration was analyzed based on the method reported by Sakurai et al. (1977). 0.5 g dry weight of sample was hydrolyzed by 2 ml of concentrated sulfuric acid (98%) at room temperature for 24 h. The mixture was diluted to 1 N of sulfuric acid solution (18.3× dilution by volume) then autoclaved at 121 °C for 15 min. Then it was neutralized with NaOH to pH 7 and further diluted to 100 ml. The glucosamine was determined as follows: 1 ml of the above sample solution was transferred into a test tube. 1 ml acetyl acetone reagent (4% (v/v) acetyl acetone in 1.25 N NaCO_3_) was added then incubated at 100 °C for 20 min. After cooling down to room temperature, 6 ml of absolute ethanol was added and then mixed gently. 1 ml of Ehrlich reagent (1.6 g of N-N dimethyl-*p*-aminobenzaldehyde was added into 60 ml solution containing 50:50 (v/v) of absolute ethanol: concentrated HCl) was added. The mix was incubated at 65 °C for 10 min and the absorbance value was determined at 530 nm.

### 2.9 HPLC

The amounts of sugars were quantified by HPLC. Prior to HPLC analysis, all samples and standards were filtered using Whatman GD/X syringe filters (GF/C 25 mm filter diameter/1.2 μm pore size; Whatman International Ltd., Banbury, UK). Monosaccharides (arabinose, galactose, glucose and xylose) were analyzed using Dionex ICS-3000 Reagent-FreeTM Ion Chromatography equipped with Dionex ICS-3000 system, electrochemical detection using ED 40 and computer controller. The CarboPacTM PA 20 column (3 × 150 mm) was used and the mobile phase was 10 mM NaOH with a flow rate of 0.5 mL/min. The injection volume was 10 μl and the column temperature was 30 °C.

## 3 Results and discussion

### 3.1 Moisture content

Wheat straw has been widely used in solid state fermentations for the production of cellulase and a broad range of moisture contents from 50% to 86% has been explored (Table 1). In this study, we investigated a relatively high moisture range in order to accelerate the growth of *A. niger* and cellulase production. Water to wheat straw ratios ranging from 5:1 to 9:1 (w/w) were examined. The biomass coverage and spore formation on the substrate surface were positively associated with the increase in moisture content, indicating that the higher the moisture, the higher the fungal growth rates were within the moisture range 85.1–91.1%. In the experiments with 8:1 and 9:1 water to wheat straw ratios, spores fully covered the substrate after culturing for 5 days. These results agree with similar data using *A. niger* on wheat straw, sugarcane bagasse and soybean bran (Delabona et al., 2013; Bansal et al., 2012; Gutierrez-Correa et al., 1999), where the culture times of 4–5 days were normally applied (Table 1). It was reported that a higher moisture content was not favorable for fungal growth due to the limitation of oxygen transfer at high water level SSF (Delabona et al., 2013; dos Santos et al., 2012; Gao et al., 2008). However, this study showed better fungal growth at a higher moisture content up to around 91.1%. This may be due to the high surface area when using Petri dishes with a relatively low loading ratio (2 g dry weight wheat straw per Petri dish), which overcame the oxygen transfer limitation.

**Fig. 2:**
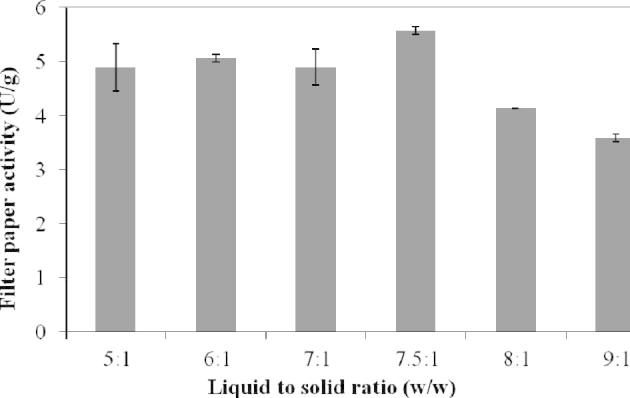
The impact of moisture content on cellulase production. Autoclaved wheat straw was used as the substrate for the fermentation by *A. niger* at 28 °C in a static incubator. The experiments were carried out with *n* = 3 and the error bars indicate standard deviation of each data set.

The cellulase accumulations from day 3 were analyzed. The highest cellulase activity was around 5.57 U/g obtained in the SSF with a water to wheat straw ratio of 7.5:1 (Fig. 2). This has been statically analyzed by two tails *t*-test with *α* = 0.05 and *n* = 5; the statistics shows that the 7.5:1 case is significantly different from the others. As SSF with 7.5:1 water to wheat straw ratio also resulted in the highest enzyme activity at day 1 (data not shown), this condition was selected for the following experiments.

### 3.2 Enzyme extraction method and culture time

In the experiments of optimizing moisture content, the enzyme mixture was extracted by mixing fermented mash with the buffer solution using a magnetic stirrer. The enzyme activity was 5.57 U/g which was relatively low compared with data reported in the literature (Bansal et al., 2012; Kang et al., 2004). An improved enzyme extraction method was then explored to optimize the enzyme extraction. In the revised method, the fermented mash and buffer mixture were blended for 10 s before being stirred. The amounts of cellulase recovered at day 3 reached 9.51 U/g. This was 71% higher than that obtained by using the enzyme extraction method without blending. The improvement can probably be attributed to the effective extraction of cellulase by mechanical force which provides open accessibility of the structure and which results in the release of enzymes into the buffer solution. The impact of the extraction method on the enzyme recovery yield was also investigated by Dhillon et al. (2012), in which extractions using three shaking conditions, wrist action shaker, incubation shaker and vortex shaker, were compared. The wrist action shaker led to an increase of cellulase activity by 16% and 29% in comparison with incubation shaker and vortex shaker respectively. This confirmed that the amount of enzyme recovered could be affected by the extraction method.

**Fig. 3:**
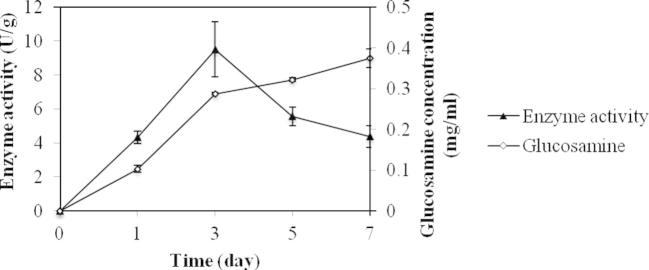
The time courses of glucosamine and enzyme activity produced over the seven days period of the experiment. Autoclaved wheat straw was used as the substrate for the fermentation by *A. niger* at 28 °C in a static incubator. The liquid to solid ratio of the fermentation was 7.5:1. The experiments were carried out with *n* = 3 and the error bars indicate standard deviation of each data set.

The time course of glucosamine concentration was determined to indicate the biomass concentration of *A. niger*, as glucosamine was reported to have a positive correlation with the fungal cell growth (Hsieh et al., 2007). As shown in Fig. 3, glucosamine concentration increased fast in the first 3 days, then slowed down. After 7 days of culturing, the glucosamine concentration was still increasing indicating that *A. niger* still grew after 7 days. The cellulase accumulation reached a maximum at day 3 (9.51 U/g). After that, the cellulase activity declined and levelled off after day 5. This is in agreement with experiments reported by several other researchers (Delabona et al., 2013).

The addition of starch in solid state fungal fermentation shortened the culture time by 5–6 days (data not shown). In liquid fermentation, glucose released from hydrolysis of starch may repress induction of cellulase gene expression (Rajmane and Korekar, 2012). However, in solid state fermentation of *A. niger*, repression of cellulase activity by starch was not observed (Fig. 3). Moreover, it was reported that the addition of starch in solid state fermentation of *Aspergillus* sp. slightly improved cellulase production (Liang et al., 2012).

### 3.3 Wheat straw modification

In the solid state fermentations, the wheat straw was autoclaved before the inoculation. The autoclave not only sterilized the substrate, but also modified the wheat straw morphology, which could be considered as a mild hydrothermal pre-treatment of the wheat straw. In this study, two other wheat straw modification methods, dilute acid and acid soaking, were investigated with the aim to improve fungal growth and cellulase production. These results were then compared with fermentations using autoclaved wheat straw and crude (non-modified) wheat straw.

Two acids, sulfuric acid and hydrochloride acid were first tested for the diluted acid modification at concentrations of 1%, 2% and 3% (w/v). Compared with hydrochloride acid, sulfuric acid led to slightly higher enzyme production (data not shown). Although 1% acid concentration was the best condition in term of cellulase production, there was no significant difference between these three concentrations (data not shown). 1% sulfuric acid was then selected for the following experiments.

**Fig. 4:**
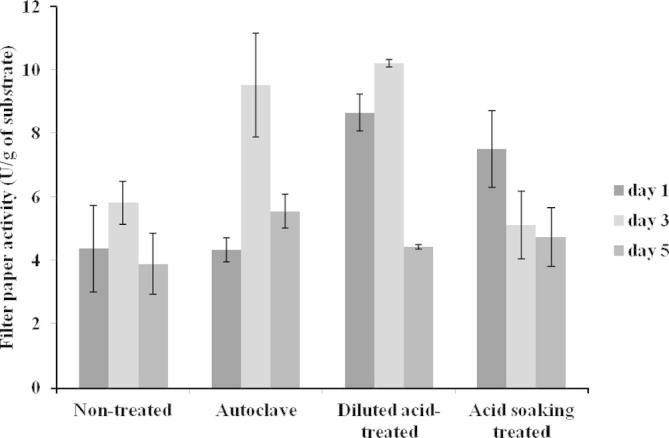
Effect of different modification methods on cellulase productions at day 1 (in black), day 3 (in light gray) and day 5 (in dark gray). The modified and non-treated wheat straw were fermented by *A. niger* with a liquid to solid ratio of 7.5:1 at 28 °C in a static incubator*.* The experiments were carried out with *n* = 6 and the error bars indicate standard deviation of each data set.

As shown in Table 2, the dilute acid and acid soaking modified wheat straw resulted in high enzyme activity at day 1. This may be due to the dilute acid and acid soaking removing the hemicellulose (Ibbett et al., 2011) exposing cellulose to *A. niger*. The induction of cellulase by wheat straw was a fast process in liquid culture, taking around 6 h (Delmas et al., 2012). Although the fermentation condition was different to liquid culture, the induction of cellulase in solid state fermentation could be fast as well. Therefore, by the first day, *A. niger* excreted higher amount of cellulase than fermentations using autoclave modified wheat straw and non-treated wheat straw. However, both dilute acid and acid soaking modified wheat straw led to low cellulase at day 5. In the dilute acid experiment, the enzyme activity dropped to 4.43 U/g at day 5, which was only 43% of the peak cellulase activity at day 3. The reduction in cellulose content and nutrient supplement may be the cause of the decrease of the cellulase activity (Singh et al., 2011). Surprisingly, *A. niger* was able to produce a significant amount of cellulase (5.83 U/g) using crude wheat straw (Table 2). This indicated that a cost effective biorefining process could be developed based on only crude wheat straw For the dilute acid-modified wheat straw, the highest cellulase activity was 10.2 U/g at day 3 (Table 2, Fig. 4). It showed that cellulase produced from the dilute acid-modified wheat straw is significantly different from the non-treated and acid soaking-modified wheat straw. However, there was no significant difference between the dilute acid-modified wheat straw and autoclaved wheat straw. Therefore, here we used the autoclaved wheat straw as the substrate due to its relatively simple operation.

Modifying the lignocellulosic materials by acid or alkali before the fungal fermentation was also explored by other researchers as a way to improve cellulase production. Bansal et al. (2012) modified wheat straw with 1% (v/v) sulfuric acid, leading to 19.2 U/g FPase from solid stage fermentations using *A. niger* NS-2. This was 8 times higher than that using the autoclaved wheat straw. When 1% (w/v) NaOH pre-treatment was used, 30.6 U/g FPase was obtained. Singh et al. (2011) investigated microwave-alkali modification method on rice straw and rice hull. Fermentations of the modified substrate by *A. heteromorphus* led to 14.1 and 8.2 U/g FPase on rice straw and rice hull, respectively.

### 3.4 Impact of additional nutrients

**Fig. 5:**
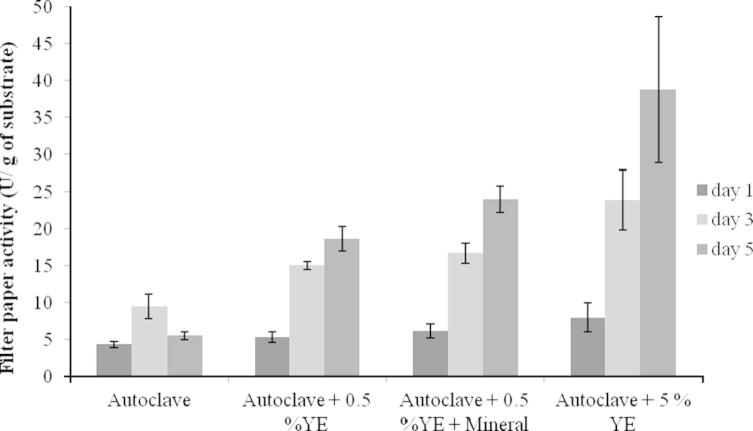
Effect of additional nutrients on cellulase production on day 1 (in black), day 3 (in light gray) and day 5 (in dark gray). The autoclaved wheat straw with or without additional nutrient(s) were fermented by *A. niger* with a liquid to solid ratio of 7.5:1 at 28 °C in a static incubator*.* The experiments were carried out with *n* = 9 and the error bars indicate standard deviation of each data set.

To further improve cellulase generation, 0.5% yeast extract (YE) was added to wheat straw to investigate the impact of adding extra nutrients on enzyme production. As shown in Table 3 and Fig. 5, the cellulase activities increased to 15.1 and 18.6 U/g after 3 and 5 days culture, respectively. More promisingly, when a mineral solution was added, the cellulase activities achieved 16.7 and 24.0 U/g after 3 and 5 days culture, respectively. When 5% YE was added, a cellulase activity of 38.8 U/g was obtained. This is in agreement with various studies that the addition of nutrients could improve cellulase production (Gao et al., 2008; Kachlishvili et al., 2006). However, Ncube et al. (2012) cultured *A. niger* FGSCA733 on a *Jatropha curcas*-based substrate. It was found that the addition of nitrogen sources did not enhance cellulase production. *A. niger* was found to grow on a medium containing YE and minerals (0.5%, in a liquid culture), but the cellulase activity was only 0.15 ± 0.01 U/ml after 5 days culture.

### 3.5 Wheat straw hydrolysis

**Fig. 6:**
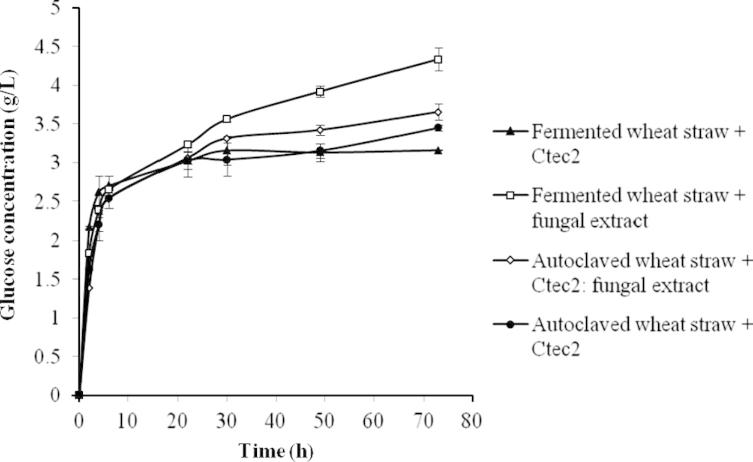
Enzyme hydrolysis of fermented wheat straw using commercial enzyme (), fermented wheat straw using fungal extract (), autoclaved wheat straw using mixture of commercial enzyme and fungal extract () and autoclaved wheat straw using commercial enzyme (), expressed as glucose released from wheat straw after enzymatic hydrolysis at 50 °C for 73 h. The experiments were carried out with *n* = 3 and the error bars indicate standard deviation of each data set.

After the solid state fungal fermentation, the fungal extract (enzyme solution) was collected and used as the cellulase solution for the enzymatic hydrolysis of the fermented wheat straw, as shown in Fig. 1. Fig. 6 presents the glucose concentration profiles of the wheat straw hydrolysis catalyzed by both the commercial cellulase Ctec2 (Novozymes) and the fungal extract (from the SSF using autoclaved wheat straw, 0.5% YE with mineral). Two substrates, fermented wheat straw and autoclaved wheat straw before the fungal fermentations were compared in order to investigate the impact of SSF itself. In the first 22 h, no significant difference was observed between these experiments. Glucose was released with a fast speed of around 0.4 g/L/h at the first 6 h. After 22 h the hydrolysis using Ctec2 stopped glucose generation, resulting in around 3.13 g/L glucose. Meanwhile, the hydrolyses using the fungal extract and mixture of fungal extract /Ctec2 continued until around 4.3 g/L (using fermented wheat straw) and 3.6 g/L (using autoclaved wheat straw) glucose were liberated into the hydrolysates. These results suggested that although the fungal extract had a lower detected cellulase activity, it performed the same or even better than the Ctec2 solution.

The endoglucanase, exoglucanase, β-glucosidase and xylanase activities of the fungal extract and the Ctec2 solution were analyzed. The activities were 5.70 ± 0.21, 1.31 ± 0.05, 5.34 ± 0.13 and 21.8 ± 1.77 IU/ml (fungal extract) and 8.35 ± 0.48, 0.45 ± 0.10, 5.25 ± 0.09 and 17.7 ± 0.64 IU/ml (the Ctec2 solution), respectively. Except endoglucanase, all of the enzyme activities in the fungal extract were higher than the Ctec2 solution. The exoglucanase in the fungal extract was nearly 2-fold higher than in the Ctec2 solution. The total protein analysis showed that the fungal extract contained around 1.82 ± 0.05 mg protein/mL, while the diluted Ctec2 solution contained only 0.90 ± 0.06 mg protein/mL, indicating that the fungal extract contained other enzymes than cellulase. Another possible explanation could be that the fungal extract was freshly produced and was secreted by the *A. niger* grown on the wheat straw. The enzyme composition of the fungal extract might then be tailored for functionality against wheat straw.

The glucose production yield in the hydrolysis using the fungal extract and fermented wheat straw was only 0.10 g glucose/g wheat straw, equating to 23.3% glucose hydrolysis yield. The glucose hydrolysis yield was relatively low in comparison with that obtained from dilute acid treatment. However, it was close to that reported in similar biological pre-treatment processes, in which around 20% glucose hydrolysis yield was normally obtained (Dias et al., 2010; Wan and Li, 2011). After the enzyme hydrolysis, a significant amount of solid residue remained, indicating that the glucose hydrolysis yield could be further improved.

## 4 Conclusion

A novel solid-state fungal fermentation-based biorefining strategy was developed to convert wheat straw into a fermentable sugar. *A. niger* was firstly cultured on the wheat straw for production of enzymes, followed by the hydrolysis of fermented wheat straw using the fungal extract. Dilute acid modification of the wheat straw improved cellulase production to 10.2 U/g, which was increased to 24.0 U/g by adding yeast extract and minerals. Wheat straw hydrolysis using the fungal extract and fermented wheat straw resulted in 4.3 g/L glucose. Compared with commercial cellulase, the freshly-prepared fungal extract released 19% higher glucose under the same hydrolysis condition.

## Figures and Tables

**Table 1:** Cellulase production from lignocellulosic feedstocks.

Strain	Substrate	Moisture content (%)	Time (day)	Enzyme activity (U/g)	References
*Aspergillus fumigatus* P40M2	Soybean bran, wheat bran	60	5	2.4–5.0	Delabona et al. (2013)
*Aspergillus heteromorphus*	Rice straw, rice hull	70	12	8.2–14.1	Singh et al. (2011)
*Aspergillus niger*	Sugarcane bagasse	80	5	5.6	Gutierrez-Correa et al. (1999)
*Aspergillus niger* KK2 mutant (KFCC 11285)	Rice straw	65	4	19.5	Kang et al. (2004)
*Aspergillus niger* NS2	Wheat straw	60	4	2.8–30.6	Bansal et al. (2012)
*Aspergillus niger* P47C3	Orange bagasse, soybean bran, wheat bran	50–70	5	0.9–5.6	Delabona et al. (2013)
*Aspergillus terreus*	Rice straw	86	7	7.2	Narra et al. (2012)
*Aspergillus tubingensis* JP-1	Wheat straw	85	8	0.67	Pandya and Gupte (2012)
Various white rot basidiomycetes	Wheat straw	81.3	10–14	12.9–46.4	Kachlishvili et al. (2006)
*Aspergillus niger* N402	Wheat straw	89.5	5	5.6–24.0	This study

**Table 2:** Effect of different modification methods on cellulase production. Filter paper activity (U/g) measured on day 1, 3 and 5. The modified and non-treated wheat straw were fermented by *A. niger* with a liquid to solid ratio of 7.5:1 at 28 °C in a static incubator*.*

Time (day)	Non-treated	Autoclave	Diluted acid	Acid soaking
0	0 ± 0	0 ± 0	0 ± 0	0 ± 0
1	4.37 ± 1.37	4.33 ± 0.38	8.65 ± 0.58	7.51 ± 1.20
3	5.83 ± 0.68	9.51 ± 1.64	10.2 ± 0.13	5.11 ± 1.08
5	3.89 ± 0.96	5.55 ± 0.54	4.43 ± 0.07	4.73 ± 0.93

**Table 3:** Effect of additional nutrients on cellulase production. Filter paper activity (U/g of dry wheat straw) measured on day 1, 3 and 5. The autoclaved wheat straw, with or without additional nutrient(s) was fermented by *A. niger* with a liquid to solid ratio of 7.5:1 at 28 °C in a static incubator.

Time (day)	Autoclave	Autoclave + 0.5% YE	Autoclave + 0.5% YE + mineral	Autoclave + 5% YE
0	0 ± 0	0 ± 0	0 ± 0	0 ± 0
1	4.33 ± 0.38	5.37 ± 0.70	6.14 ± 0.96	7.96 ± 1.97
3	9.51 ± 1.64	15.1 ± 0.51	16.7 ± 1.33	23.9 ± 4.10
5	5.55 ± 0.54	18.6 ± 1.64	24.0 ± 1.76	38.8 ± 9.85
